# Association of Twice-Daily Radiotherapy With Subsequent Brain Metastases in Adults With Small Cell Lung Cancer

**DOI:** 10.1001/jamanetworkopen.2019.0103

**Published:** 2019-05-17

**Authors:** Haiyan Zeng, Rui Li, Chen Hu, Guoqin Qiu, Hong Ge, Huiming Yu, Kaixian Zhang, Miaomiao Hu, Peng Zeng, Dan Xiao, Chuanwang Miao, Chuqing Wei, Meng Ni, Jingyi Shen, Hui Li, Jinbo Yue, Heming Lu, Bingjie Fan, Hui Zhu, Xudong Hu, Feng-Ming (Spring) Kong, Jinming Yu, Shuanghu Yuan

**Affiliations:** 1 Department of Radiation Oncology, Shandong Cancer Hospital Affiliated to Shandong University, Jinan, Shandong, China; 2 Shandong Academy of Medical Sciences, Jinan, Shandong, China; 3 Department of Radiation Oncology, Sichuan Cancer Hospital and Institute, Sichuan Cancer Center, School of Medicine, University of Electronic Science and Technology of China, Chengdu, Sichuan, China; 4 Sidney Kimmel Comprehensive Cancer Center, Johns Hopkins University School of Medicine, Baltimore, Maryland; 5 Department of Radiation Oncology, Zhejiang Cancer Hospital, Hangzhou, Zhejiang, China; 6 Department of Radiation Oncology, The Affiliated Cancer Hospital of Zhengzhou University, Henan Cancer Hospital, Zhengzhou, Henan, China; 7 Key Laboratory of Carcinogenesis and Translational Research (Ministry of Education), Department of Radiation Oncology, Peking University Cancer Hospital & Institute, Beijing, China; 8 Department of Oncology, Tengzhou Central People’s Hospital, Tengzhou, Shandong, China; 9 Department of Emergency, The Second Affiliated Hospital of Nanchang University, Nanchang, Jiangxi, China; 10 Department of Oncology, Jiangxi Cancer Hospital, Nanchang, Jiangxi, China; 11 School of Medicine and Life Sciences, University of Jinan-Shandong Academy of Medical Sciences, Jinan, Shandong, China; 12 Shandong University, Jinan, Shandong, China; 13 Department of Oncology, The First Affiliated Hospital of Henan University, Kaifeng, Henan, China; 14 Department of Radiation Oncology, People’s Hospital of Guangxi Zhuang Autonomous Region, Nanning, Guangxi, China; 15 Department of Radiation Oncology, Case Western Reserve University, Cleveland, Ohio

## Abstract

**Importance:**

Although thoracic twice-daily radiotherapy (TDRT) is one of the standards of care for small cell lung cancer, its association with brain metastases remains unknown.

**Objective:**

To investigate the association of TDRT vs once-daily radiotherapy (ODRT) with brain metastases after prophylactic cranial irradiation in patients with small cell lung cancer.

**Design, Setting, and Participants:**

In this multicenter cohort study, data on 778 consecutive patients with small cell lung cancer who had undergone thoracic radiotherapy (609 received ODRT and 169 received TDRT), chemotherapy, and prophylactic cranial irradiation were retrieved from the databases of 8 hospitals in China between July 1, 2003, and June 30, 2016. A 1:1 propensity score matching approach was used to control for confounding between the ODRT and TDRT groups. Confounding covariates included 8 demographic variables and 8 treatment-related covariates. Data analysis was conducted from November 1, 2017, to May 31, 2018, and reanalyzed for revision.

**Exposures:**

The ODRT group received 50 to 66 Gy given in 25 to 33 fractions. The TDRT group received 45 Gy given in 30 fractions.

**Main Outcomes and Measures:**

The primary end point was brain metastases. Secondary end points included progression-free survival and overall survival.

**Results:**

Of the 778 patients (median age, 55 years [interquartile range, 48-61 years]), 204 were women and 574 were men. At a median follow-up of 23.6 months (interquartile range, 14.2-38.2 months), 131 patients (16.8%) experienced brain metastases. The rate of brain metastasis at 3 years in the TDRT group was significantly higher than in the ODRT group (26.0% vs 16.9%; hazard ratio, 1.55; 95% CI, 1.06-2.26; *P* = .03). Of the 338 matched patients (169 in the ODRT group vs 169 in the TDRT group), 60 (17.8%) experienced brain metastases, with a rate at 3 years of 14.9% in the ODRT group vs 26.0% in the TDRT group (hazard ratio, 1.71; 95% CI, 1.02-2.88; *P* = .04). Progression-free survival was similar in both the whole cohort and the matched cohort. Median overall survival in the ODRT group tended to be significantly longer than in the TDRT group after matching (47.2 vs 32.8 months; hazard ratio, 1.41; 95% CI, 0.99-2.01; *P* = .06).

**Conclusions and Relevance:**

In this study, patients with small cell lung cancer who received thoracic TDRT appeared to have a higher risk of brain metastases than those who received ODRT, which supports the need for further prospective randomized clinical trials, especially in China and other parts of Asia.

## Key Points

**Question:**

Does thoracic twice-daily radiotherapy increase the risk for brain metastases after prophylactic cranial irradiation in patients with small cell lung cancer?

**Findings:**

This multicenter cohort study, involving 778 patients during the period from 2003 to 2016, revealed a significantly higher rate of brain metastasis among patients who underwent twice-daily radiotherapy than among those who underwent once-daily radiotherapy, which was further confirmed in subsequent propensity score analysis of 338 patients.

**Meaning:**

Compared with patients with small cell lung cancer who received once-daily radiotherapy, those who received twice-daily radiotherapy were more likely to experience brain metastases after prophylactic cranial irradiation.

## Teaser

This multicenter cohort study investigates the association of thoracic twice-daily radiotherapy vs once-daily radiotherapy with brain metastases after prophylactic cranial irradiation in adult Chinese patients with small cell lung cancer (SCLC).

## Introduction

Twice-daily radiotherapy (TDRT) (1.5 Gy twice daily for a total of 45 Gy [to convert gray to rad, multiply by 100]) or once-daily radiotherapy (ODRT) (1.8-2.0 Gy once daily for a total of 60-70 Gy) is recommended for small cell lung cancer in the 2018 National Comprehensive Cancer Network guideline.^1^ However, the effect of TDRT on brain metastases remains unknown.

The NCCTG (North Central Cancer Treatment Group) 89-20-52 trial reported that the rates of brain metastasis for patients receiving TDRT were numerically higher than the rates of brain metastasis for patients receiving ODRT among 154 patients with limited disease (11% vs 9%; *P* = .68).^2^ In stage IIIA or IIIB non–small cell lung cancer, hyperfractionated accelerated radiotherapy was associated with higher rates of brain metastasis than was ODRT (20% vs 13%), but the calculated time point and *P* value were not reported.^3^ One retrospective study exploring risk factors for brain metastases after prophylactic cranial irradiation in patients with small cell lung cancer reported that, compared with patients receiving ODRT, patients receiving TDRT were more likely to develop brain metastases (3-year rates, 43% with TDRT vs 21% with ODRT; hazard ratio [HR], 2.171; 95% CI, 1.111-4.243; *P* = .02).^4^ However, according to the CONVERT (Concurrent Once-daily Versus Twice-daily Radiotherapy) trial using competing risk analysis, the incidence of brain metastases was similar in each arm (5-year rates, 18.3% with TDRT vs 15.9% with ODRT; HR, 1.15; 95% CI, 0.75-1.79; *P* = .42).^5^

To further investigate the association of thoracic TDRT and ODRT with brain metastases after prophylactic cranial irradiation, we conducted this retrospective study of patients with small cell lung cancer treated at 8 institutions in China, using methods that included competing risk analysis and propensity score matching.

## Methods

**Figure 1.**
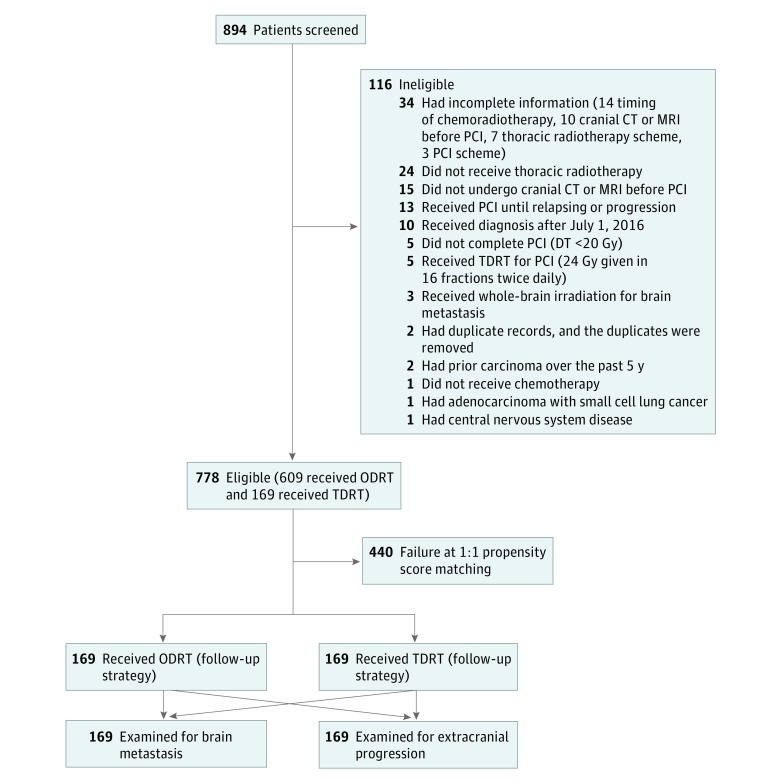
Study Participants Screened, Eligible, and Included CT indicates computed tomography; DT, total dose; MRI, magnetic resonance imaging; ODRT, once-daily radiotherapy; PCI, prophylactic cranial irradiation; and TDRT, twice-daily radiotherapy. To convert gray to rad, multiply by 100. Follow-up strategy provided in detail in the eAppendix in the Supplement.

In this multicenter cohort study, data on 778 consecutive patients with small cell lung cancer who had undergone thoracic radiotherapy (609 received ODRT and 169 received TDRT), chemotherapy, and prophylactic cranial irradiation were retrieved from the databases of 8 hospitals in China between July 1, 2003, and June 30, 2016. Criteria for eligibility for this study included the following: (1) pathologically or cytologically confirmed small cell lung cancer without a mixture of other pathologic types; (2) receipt of radiotherapy, chemotherapy, and once-daily prophylactic cranial irradiation before relapsing or progression from July 1, 2003, to June 30, 2016; (3) underwent contrast-enhanced cranial computed tomography or magnetic resonance imaging to rule out brain metastases prior to prophylactic cranial irradiation (a prior study^4^ showed that performance of computed tomography or magnetic resonance imaging before prophylactic cranial irradiation was not significantly related with brain metastases [at 3 years: computed tomography, 28% vs magnetic resonance imaging, 18%; *P* = .37], so we did not specify computed tomography or magnetic resonance imaging in this study); and (4) no evidence of prior malignant carcinoma during the past 5 years. Patients with incomplete medical records at diagnosis or treatment were excluded from this analysis (Figure 1). Disease stage was not the excluding criteria in this study because Slotman et al^6,7^ found that patients with an extensive stage of the disease also benefit from prophylactic cranial irradiation and thoracic radiotherapy. Instead, we matched and adjusted for disease stage in further analyses. This retrospective study was approved by the medical-record department of Shandong Cancer Hospital according to the institutional rules that reviewers should not take photographs, share the contents, or breach patients’ privacy. Those who disobey these rules would be revoked the reviewing permission for 3 to 6 months. We obeyed the rules strictly, and all personal information had been removed to protect patient privacy (patient informed consent was exempted). This study followed the International Society for Pharmacoeconomics and Outcomes Research (ISPOR) reporting guideline for comparative effectiveness studies according to the Strengthening the Reporting of Observational Studies in Epidemiology (STROBE) statements.^8^

The most commonly used schedules were 50 to 66 Gy given in 25 to 33 fractions once daily for ODRT and 45 Gy given in 30 fractions twice daily for TDRT (eTable 1 in the Supplement). The mainly prophylactic cranial irradiation schedule was 25 Gy given in 10 fractions once daily. Patients who received 24 Gy given in 16 fractions twice daily were excluded.

The biologically effective dose (BED) of thoracic radiotherapy was calculated according to the linear-quadratic formula^9^: BED = (*nd*){1 + [*d*/(α/β)]} – [0.693*t*/(α*T*_pot_)], where *n* is the total number of fractions delivered, *d* is the dose per fraction (in units of gray), α/β = 10, α = 0.3 Gy, *t* is the total number of days in which radiotherapy was delivered, and *T*_pot_ is the potential doubling time (5.6 days).^9,10^

Response to chemoradiotherapy was assessed with the Response Evaluation Criteria in Solid Tumors version 1.1 criteria^11^ before prophylactic cranial irradiation but was not specified in this study because an earlier study found that response was not associated with brain metastasis (at 3 years: complete response, 29% vs partial response or stable disease, 27%; *P* = .84).^4^ The detailed follow-up strategy is presented in the eAppendix in the Supplement. Additional brain radiotherapy (radiosurgery or whole-brain radiotherapy, depending on whether oligometastasis in the brain or multiple brain metastasis) and chemotherapy were adopted for patients with brain metastases but not specified in this study.

The primary end point was brain metastases confirmed by cranial imaging at any time whether neurologic symptoms (eg, headache or vomiting) were present or not. The secondary end points were progression-free survival (progression of disease at the first time in any sites confirmed by imaging) and overall survival. All end points were analyzed as time-to-event data from the start of thoracic radiotherapy to the respective events, which were subject to censoring at the last follow-up (data cutoff was November 13, 2017) if no events were observed. The brain metastases were evaluated using competing risk analysis (the Gray test for univariate analysis and the Fine-Gray model for multivariable regression^12,13^), in which death without brain metastases was treated as a competing event. Both progression-free survival and overall survival were analyzed using the Kaplan-Meier method and Cox proportional hazards regression models. Six clinically important covariates (year of diagnosis, performance status, disease stage, thoracic radiotherapy, combination of chemoradiotherapy, and timing of prophylactic cranial irradiation) were included for multiple analysis.

A 1:1 optimal propensity score–matched method was used to control confounding between the ODRT (control) and TDRT (treated) groups^14^ to essentially estimate the average treatment effect on the treated, instead of the average treatment effect on the entire sample. Propensity scores (ie, the conditional probability of receiving TDRT) were calculated using a multivariable logistic regression model. The covariates used to calculate propensity scores included 8 demographic variables (treating site, year of diagnosis, age at diagnosis, sex, performance status, smoking history, laterality, and disease stage) and 8 treatment-related covariates (surgery, combination of chemoradiotherapy, type of initial chemotherapy regimen, types of chemotherapy regimen involved, chemotherapy cycles, thoracic radiotherapy time from diagnosis to start of therapy, timing of prophylactic cranial irradiation, and dose classification of prophylactic cranial irradiation), which were summarized and compared between the TDRT and ODRT groups using χ^2^ tests, both prior to and after matching. All tests were 2-sided, and *P* < .05 was considered statistically significant. Statistical analyses were performed from November 1, 2017, to May 31, 2018, and reanalyzed for revision using IBM SPSS, version 22.0 (IBM Corp) and R, version 2.15.3 (R Project for Statistical Computing).

## Results

### Patient Characteristics

Of the 894 consecutive patients queried, 778 met the study criteria with complete medical records and were included (Figure 1). Of the 778 patients (median age, 55 years [interquartile range, 48-61 years]), 204 (26.2%) were women, 490 (63.0%) were smokers (among the 574 male patients, 478 [83.3%] were smokers), 321 (41.3%) underwent radiotherapy sequentially after 2 to 4 cycles of chemotherapy (sequential chemoradiotherapy) rather than concurrently (concurrent chemoradiotherapy) largely owing to performance status or age, 609 (78.3%) received ODRT, and the other 169 (21.7%) received TDRT at treating physicians’ discretions based on their department’s inclinations (in some departments, physicians prefer ODRT for all of their patients, while in other departments, physicians prefer TDRT) (Table 1). The median duration of thoracic radiotherapy was 64 days (interquartile range, 42-102 days).

**Table 1.** Clinical Features and Risk of BM Before Propensity Score Matching

Characteristic	Patients, No. (%) (N = 778)	BM Rate, %		Univariate		Multivariate	
		3 y	5 y	HR (95% CI)	*P* Value	HR (95% CI)	*P* Value
Treating site							
A(SD)	258 (33.2)	19.7	24.7	0.99 (0.87-1.13)	.86	No data	
B(SC)	166 (21.3)	18.6	24.8				
C(ZJ)	198 (25.4)	16.5	18.8				
D(HN)	77 (9.9)	24.7	NA				
E(BJ)	48(6.2)	16.1	NA				
F(TZ/NC/JX)	31 (4.0)	22.6	22.6				
Year of diagnosis							
2003-2010	292 (37.5)	18.0	22.0	1.03 (0.72-1.46)	.88	0.83 (0.55-1.27)	.39
2011-2016	486 (62.5)	19.6	24.2				
Age at diagnosis, y							
<60	527 (67.7)	16.9	22.3	1.20 (0.84-1.71)	.32	No data	
≥60	251 (32.3)	23.1	24.5				
Sex							
Male	574 (73.8)	19.8	25.0	1.01 (0.69-1.48)	.94	No data	
Female	204 (26.2)	18.4	22.5				
Performance status							
0	127 (16.3)	13.5	23.5	1.29 (0.86-1.95)	.22	1.25 (0.81-1.91)	.32
1	624 (80.2)	19.7	22.8				
2	27 (3.5)	20.9	NA				
Smoking history							
Yes	490 (63.0)	19.4	26.4	0.98 (0.69-1.39)	.93	No data	
No	288 (37.0)	18.4	21.4				
Laterality							
Left	380 (48.8)	18.8	24.2	0.94 (0.67-1.32)	.71	No data	
Right	398 (51.2)	18.7	21.8				
Stage							
Limited disease	684 (87.9)	17.9	21.8	0.94 (0.67-1.32)	.03	1.69 (1.03-2.77)	.04
Extensive disease	94 (12.1)	25.0	36.4				
Surgery							
Yes	44 (5.7)	12.0	19.6	0.75 (0.36-1.58)	.45	No data	
No	734 (94.3)	19.2	23.2				
Type of initial chemotherapy regimen							
Etopside-platinum	719 (92.4)	18.4	22.2	1.33 (0.76-2.33)	.32	No data	
Non–etopside-platinum	59 (7.6)	22.3	31.6				
Types of chemotherapy regimen involved, No.							
1	668 (85.9)	18.2	22.5	1.17 (0.75-1.84)	.48	No data	
≥2	110 (14.1)	21.4	25.6				
Chemotherapy cycles, No.							
<4	27 (3.5)	9.2	9.2	1.50 (0.88-2.54)	.13	No data	
4-6	710 (91.3)	18.7	23.5				
>6	41 (5.3)	25.2	25.2				
Thoracic radiotherapy time, d^a^							
≤64	393 (50.5)	18.0	22.7	1.09 (0.78-1.53)	.62	No data	
>64	385 (49.5)	19.5	23.6				
Thoracic radiotherapy							
ODRT	609 (78.3)	16.9	21.6	1.55 (1.06-2.26)	.03	1.57 (1.04-2.37)	.03
TDRT	169 (21.7)	26.0	28.1				
Combination of chemoradiotherapy							
SCRT	321 (41.3)	20.0	25.7	0.83 (0.59-1.17)	.28	0.87 (0.62-1.23)	.42
CCRT	457 (58.7)	17.8	21.1				
Timing of prophylactic cranial irradiation							
Early^b^	155 (19.9)	23.1	26.2	1.33 (0.89-2.00)	.17	1.10 (0.70-1.79)	.69
Late	623 (80.1)	17.8	22.3				
Prophylactic cranial irradiation dose classification							
Lower-standard	17 (2.2)	18.7	18.7	1.09 (0.68-1.73)	.73	No data	
Standard^c^	678 (87.1)	18.4	22.8				
Higher-standard	83 (10.7)	20.6	24.8				

Abbreviations: A(SD), Shandong Cancer Hospital; BM, brain metastasis; B(SC), Sichuan Cancer Hospital; C(ZJ), Zhejiang Cancer Hospital; CCRT, concurrent chemoradiotherapy; D(HN), Henan Cancer Hospital; E(BJ), Peking University Cancer Hospital & Institute; F(TZ/NC/JX), Tengzhou Central People's Hospital, The Second Affiliated Hospital of Nanchang University, Jiangxi Cancer Hospital; HR, hazard ratio; NA, not applicable; ODRT, once-daily radiotherapy; SCRT, sequential chemoradiotherapy; TDRT, twice-daily radiotherapy.

^a^ Thoracic radiotherapy time was divided into 2 categories by median time.

^b^ Early indicates receiving prophylactic cranial irradiation before the end of chemoradiotherapy.

^c^ Standard indicates 25 Gy given in 10 fractions or 30 Gy given in 10 to 15 fractions (to convert gray to rad, multiply by 100).

### Risk of Brain Metastases and Survival Analyses

**Figure 2.**
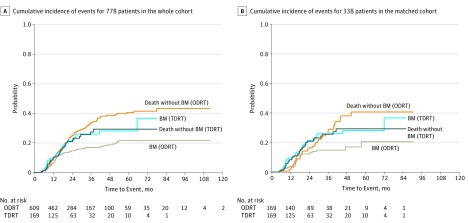
Cumulative Incidence of Events A, Cumulative incidence of brain metastasis (BM) and death without BM among the total cohort (N = 778). B, Cumulative incidence of BM and death without BM among the matched cohort (n = 338). The incidence of BM was significantly higher in the twice-daily radiotherapy (TDRT) group than in the once-daily radiotherapy (ODRT) group both before (*P* = .20) and after (*P* = .04) matching. The incidence of death without BM was not significantly different between the TDRT group and the ODRT group either before (*P* = .20) or after (*P* = .83) matching.

Of the 778 patients, 131 (16.8%) developed brain metastases at a median follow-up time of 23.6 months (interquartile range, 14.2-38.2 months), with a 3-year rate of 18.5% (95% CI, 15.6%-21.7%). Univariate analyses showed that, compared with patients treated with ODRT, those treated with TDRT were more likely to experience brain metastases after prophylactic cranial irradiation (3-year rate, 16.9% in the ODRT group vs 26.0% in the TDRT group; HR, 1.55; 95% CI, 1.06-2.26; *P* = .03) (Figure 2A). Disease stage (HR, 1.64; 95% CI, 1.03-2.62; *P* = .03) was significantly associated with brain metastases (Table 1). Multivariable analysis confirmed that patients who received TDRT (HR, 1.57; 95% CI, 1.04-2.37; *P* = .03) or those with extensive disease (HR, 1.69; 95% CI, 1.03-2.77; *P* = .04) had higher rates of brain metastasis. No significant difference between ODRT and TDRT was observed in overall survival (HR, 1.15; 95% CI, 0.88-1.50; *P* = .31) or progression-free survival (HR, 1.10; 95% CI, 0.87-1.37; *P* = .44). Multiple analysis showed that patients with poorer performance status (overall survival: HR, 1.38; 95% CI, 1.03-1.83; *P* = .03; progression-free survival: HR, 1.23; 95% CI, 0.97-1.56; *P* = .08) or those who underwent prophylactic cranial irradiation before the end of chemoradiotherapy (overall survival: HR, 1.37; 95% CI, 1.05-1.78; *P* = .02; progression-free survival: HR, 1.34; 95% CI, 1.07-1.68; *P* = .01) had poorer survival. In addition, patients who received a diagnosis in earlier years (2003-2010) or with extensive disease experienced shorter progression-free survival (Table 2).

**Table 2.** Survival Risk Using Multivariate Cox Regression Analysis^a^

Variable	Overall Survival		Progression-Free Survival	
	HR (95% CI)	*P* Value	HR (95% CI)	*P* Value
Year of diagnosis	0.82 (0.65-1.04)	.11	0.79 (0.64-0.97)	.03
Performance status	1.38 (1.03-1.83)	.03	1.23 (0.97-1.56)	.08
Disease stage	1.27 (0.90-1.79)	.17	1.45 (1.09-1.93)	.01
Thoracic radiotherapy	1.13 (0.86-1.50)	.38	1.13 (0.89-1.43)	.32
Combination of chemoradiotherapy	0.89 (0.71-1.11)	.30	0.92 (0.76-1.11)	.39
Timing of PCI	1.37 (1.05-1.78)	.02	1.34 (1.07-1.68)	.01

Abbreviations: HR, hazard ratio; PCI, prophylactic cranial irradiation.

^a^ Data on 778 consecutive patients with small cell lung cancer who had undergone thoracic radiotherapy.

Propensity score matching was used to further evaluate the role of TDRT and ODRT. A total of 338 patients were matched successfully. As shown in Table 3, patients’ clinical features, excluding treatment site, were balanced between the TDRT and ODRT groups after matching.

**Table 3.** Clinical Features Before and After Propensity Score Matching

Characteristic	Before Propensity Score Matching, No. (%)			After Propensity Score Matching, No. (%)		
	ODRT (n = 609)	TDRT (n = 169)	*P* Value	ODRT (n = 169)	TDRT (n = 169)	*P* Value
Treating site						
A(SD)	185 (30.4)	73 (43.2)	<.001	45 (26.6)	73 (43.2)	<.001
B(SC)	132 (21.7)	34 (20.1)		24 (14.2)	34 (20.1)	
C(ZJ)	188 (30.9)	10 (5.9)		36 (21.3)	10 (5.9)	
D(HN)	52 (8.5)	25 (14.8)		32 (18.9)	25 (14.8)	
E(BJ)	29 (4.8)	19 (11.2)		17 (10.1)	19 (11.2)	
F(TZ/NC/JX)	23 (3.8)	8 (4.7)		15 (8.9)	8 (4.7)	
Year of diagnosis						
2003-2010	264 (43.3)	28 (16.6)	<.001	30 (17.8)	28 (16.6)	.77
2011-2016	345 (56.7)	141 (83.4)		139 (82.2)	141 (83.4)	
Age at diagnosis, y						
<60	421 (69.1)	106 (62.7)	.12	101 (59.8)	106 (62.7)	.58
≥60	188 (30.9)	63 (37.3)		68 (40.2)	63 (37.3)	
Sex						
Male	453 (74.4)	121 (71.6)	.47	119 (70.4)	121 (71.6)	.81
Female	156 (25.6)	48 (28.4)		50 (29.6)	48 (28.4)	
Performance status						
0	113 (18.6)	14 (8.3)	.006	11 (6.5)	14 (8.3)	.78
1	476 (78.2)	148 (87.6)		152 (89.9)	148 (87.6)	
2	20 (3.3)	7 (4.1)		6 (3.6)	7 (4.1)	
Smoking history						
Yes	388 (63.7)	102 (60.4)	.42	89 (52.7)	102 (60.4)	.15
No	221 (36.3)	67 (39.6)		80 (47.3)	67 (39.6)	
Laterality						
Left	296 (48.6)	84 (49.7)	.80	73 (43.2)	84 (49.7)	.23
Right	313 (51.4)	85 (50.3)		96 (56.8)	85 (50.3)	
Stage						
Limited disease	540 (88.7)	144 (85.2)	.22	143 (84.6)	144 (85.2)	.88
Extensive disease	69 (11.3)	25 (14.8)		26 (15.4)	25 (14.8)	
Surgery						
Yes	38 (6.2)	6 (3.6)	.18	4 (2.4)	6 (3.6)	.52
No	571 (93.8)	163 (96.4)		165 (97.6)	163 (96.4)	
Type of initial chemotherapy regimen						
Etopside-platinum	556 (91.3)	163 (96.4)	.03	157 (92.9)	163 (96.4)	.15
Non–etopside-platinum	53 (8.7)	6 (3.6)		12 (7.1)	6 (3.6)	
Types of chemotherapy regimen involved, No.						
1	520 (85.4)	148 (87.6)	.47	147 (87.0)	148 (87.6)	.87
≥2	89 (14.6)	21 (12.4)		22 (13.0)	21 (12.4)	
Chemotherapy cycles, No.						
<4	20 (3.3)	7 (4.1)	.47	11 (6.5)	7 (4.1)	.63
4-6	554 (91.0)	156 (92.3)		152 (89.9)	156 (92.3)	
>6	35 (5.7)	26 (3.6)		6 (3.6)	26 (3.6)	
Thoracic radiotherapy time, d^a^						
≤64	313 (51.4)	80 (47.3)	.35	81 (47.9)	80 (47.3)	.91
>64	296 (48.6)	89 (52.7)		88 (52.1)	89 (52.7)	
Combination of chemoradiotherapy						
SCRT	231 (37.9)	90 (53.3)	<.001	96 (56.8)	90 (53.3)	.51
CCRT	378 (62.1)	79 (46.7)		73 (43.2)	79 (46.7)	
Timing of prophylactic cranial irradiation						
Early^b^	122 (20.0)	33 (19.5)	.89	26 (15.4)	33 (19.5)	.32
Late	487 (80.0)	136 (80.5)		143 (84.6)	136 (80.5)	
Prophylactic cranial irradiation dose classification						
Lower-standard	15 (2.5)	2 (1.2)	.04	3 (1.8)	2 (1.2)	.73
Standard^c^	521 (85.6)	157 (92.9)		153 (90.5)	157 (92.9)	
Higher-standard	73 (12.0)	10 (5.9)		13 (7.7)	10 (5.9)	

Abbreviations: A(SD), Shandong Cancer Hospital; B(SC), Sichuan Cancer Hospital; C(ZJ), Zhejiang Cancer Hospital; CCRT, concurrent chemoradiotherapy; D(HN), Henan Cancer Hospital; E(BJ), Peking University Cancer Hospital & Institute; F(TZ/NC/JX), Tengzhou Central People’s Hospital, The Second Affiliated Hospital of Nanchang University, Jiangxi Cancer Hospital; ODRT, once-daily radiotherapy; SCRT, sequential chemoradiotherapy; TDRT, twice-daily radiotherapy.

^a^ Thoracic radiotherapy time was divided into 2 categories by median time.

^b^ Early: receiving prophylactic cranial irradiation before the end of chemoradiotherapy.

^c^ Standard: 25 Gy given as 10 fractions or 30 Gy given as 10 to 15 fractions.

After a median follow-up of 25.9 months (interquartile range, 15.7-35.2 months), 60 of the 338 matched patients (17.8%) developed brain metastases, with a 3-year rate of 14.9% for the ODRT group and 26.0% for the TDRT group (HR, 1.71; 95% CI, 1.02-2.88; *P* = .04) (eTable 2 in the Supplement; Figure 2B). A total of 123 patients in the matched cohort (36.4%) died, with median overall survival of 47.2 months in the ODRT group and 32.8 months in the TDRT group (HR, 1.41; 95% CI, 0.99-2.01; *P* = .06) (eFigure in the Supplement). A total of 188 patients in the matched cohort (55.6%) experienced progression, with median progression-free survival of 20.1 months in the ODRT group vs 18.8 months in the TDRT group (HR, 1.16; 95% CI, 0.87-1.55; *P* = .30) (eFigure in the Supplement).

For the matched cohort, the median BED was lower in the TDRT group than in the ODRT group (43.1 vs 51.8 Gy; *P* < .001). The period from the start of any therapy to the end of radiotherapy (SER) was shorter in the TDRT group than in the ODRT group (median, 81 vs 108 days; *P* < .001). Neither BED nor SER was associated with brain metastases, progression-free survival, or overall survival. The differences in overall survival between the ODRT and TDRT groups became more obvious after adjusting for BED and SER (HR, 1.69; 95% CI, 1.05-2.71; *P* = .03) (eTable 3 in the Supplement).

Among the 5 excluded patients who received twice-daily prophylactic cranial irradiation (24 Gy given in 16 fractions twice daily) (screened among the 894 patients but not included among the 778 patients) (Figure 1), 3 (60%) experienced brain metastases. One of these 5 patients also received thoracic TDRT; he developed brain metastases. In addition, the asymptomatic brain metastasis ratio was 65.6% in the ODRT group vs 60.5% in the TDRT group (*P* = .59) before matching and 57.1% in the ODRT group vs 60.5% in the TDRT group (*P* = .80) after matching.

### Other Prognostic Factors and Subgroup Analyses

Subgroup analyses based on disease stage, year of diagnosis, and timing of prophylactic cranial irradiation were performed among the 338 matched patients. Patients who received TDRT showed higher risks of developing brain metastases when adjusting by disease stage (HR, 1.71; 95% CI, 1.02-2.87; *P* = .04). Possibly because of reduced sample size and statistical power, the rates of brain metastasis in the TDRT group were only numerically higher in either limited-stage or extensive-stage subgroups (eTable 4 in the Supplement).

For patients who received a diagnosis of small cell lung cancer in recent years (2011-2016), no significant difference in the rate of brain metastasis was observed (HR, 1.47; 95% CI, 0.80-2.68; *P* = .21). Although for those who received a diagnosis in earlier years (2003-2010), the rate of brain metastasis was significantly higher in the TDRT group (HR, 3.05; 95% CI, 1.09-8.53; *P* = .03). After adjusting for year of diagnosis, TDRT continued to be significantly associated with higher risks of brain metastasis (HR, 1.78; 95% CI, 1.05-3.00; *P* = .03) (eTable 5 in the Supplement).

In addition, the rate of brain metastasis in earlier or recent years by stratum of ODRT and TDRT was analyzed to see whether time itself would affect the results. It showed that, for patients with ODRT, the rate of brain metastasis of those who received a diagnosis in earlier years was not significantly different from those who received a diagnosis in recent years (HR, 0.97; 95% CI, 0.37-2.55; *P* = .95). Although for patients with TDRT, the rate of brain metastasis was significantly higher for those who received a diagnosis in earlier years than for those who received a diagnosis recently (HR, 0.46; 95% CI, 0.22-0.96; *P* = .04). After adjustment for ODRT and TDRT, time of diagnosis was not significantly associated with risks of brain metastases (HR, 0.61; 95% CI, 0.34-1.10; *P* = .10) (eTable 6 in the Supplement).

For patients with late prophylactic cranial irradiation, the rates of brain metastasis tended to be higher in the TDRT group but marginally significant (HR, 1.81; 95% CI, 0.99-3.31; *P* = .05), and the rates of brain metastasis were not significantly different among those who received early prophylactic cranial irradiation (HR, 1.39; 95% CI, 0.49-3.99; *P* = .54). Patients treated with TDRT showed a trend of higher, but marginally significant, risks of developing brain metastases when adjusting by the timing of prophylactic cranial irradiation (HR, 1.71; 95% CI, 1.01-2.89; *P* = .05) (eTable 7 in the Supplement).

## Discussion

This multicenter study revealed that, compared with ODRT, TDRT was associated with higher incidences of brain metastases, as shown in both multivariable analysis based on the whole cohort of 778 patients and the propensity score–matched cohort of 338 patients. Additional subgroup analyses also suggest that such disparity may be independent of disease stages and timing of prophylactic cranial irradiation.

Our analysis therefore suggests that ODRT may be superior to TDRT in controlling brain metastases in patients with small cell lung cancer, with multiple unverified, hypothesis-generating underlying mechanisms. One of the possibilities may be related to the impairment of the blood–spinal cord barrier or the blood-brain barrier. Irradiation disrupts the blood–spinal cord barrier, with an associated increase in vascular permeability in the early period after irradiation (24 hours).^15,16^ Because spinal cord tissue and vascular endothelial cells are considered late reaction tissues with a very slow rate of turnover,^17,18^ the repair of sublethal injury in spinal cord tissue and vascular endothelial cells appears to be somewhat longer.^3^ Twice-daily radiotherapy involves the delivery of the target dose in a shorter time interval between fractions and provides healthy tissues with less time to repair sublethal radiation damage, which leads to an accumulation of incomplete repair and results in an asymptomatic biological response characterized by sequential physiological changes in the thoracic spinal cord.^16,19,20^ Sublethal injury with protractedly less time for repair leads to more severe disruption of the permeability of the thoracic blood–spinal cord barrier and results in more transmigration of residual tumor cells during TDRT compared with ODRT. The transmigrated tumor cells metastasize to the brain along with cerebrospinal fluid, form metastatic niches, and generate colonization in the brain months later.^21^

This inference can also be supported by the subgroup analyses spanning 13 years, during which time significant advancements in radiotherapy technique and supportive care have been made; thus, we have adjusted for the year of diagnosis to minimize the potential confounding in our study. It showed that, for patients who received a diagnosis of small cell lung cancer in earlier years, the rate of brain metastasis was obviously higher in the TDRT group, which remained significant after adjusting years. We even compared the rate of brain metastasis in earlier and recent years by stratum of ODRT and TDRT to see the association of time itself. Again, the results showed that, for patients undergoing TDRT, the rate of brain metastasis was significantly higher among those who received a diagnosis in earlier years. However, after adjustment for ODRT and TDRT, time of diagnosis was not significantly associated with risk of brain metastases. In other words, TDRT was more detrimental in earlier years. The improved radiologic techniques and better supportive care over the years might minimize the difference of brain metastases between ODRT and TDRT. Thus, improved techniques can decrease the level of injury to the thoracic blood–spinal cord barrier and mask the difference. This finding may also explain the results of studies from Western countries, such as the CONVERT study, in which there was no difference in the rates of brain metastasis between patients who underwent TDRT and those who underwent ODRT.^5^

In addition, the incidence of brain metastases among patients who received prophylactic cranial irradiation twice daily was much higher than among patients who received once-daily prophylactic cranial irradiation, which further indicated that irradiating the whole brain twice daily would more obviously injure the blood-brain barrier. In addition, data from other studies, such as the RTOG (Radiation Therapy Oncology Group) 0212 on prophylactic cranial irradiation, also showed that the rate of brain metastasis at 1 year was 10.6% in the twice-daily arm (36 Gy given in 24 fractions) vs 6.2% in the once-daily arm (36 Gy given in 18 fractions) (total, 21% in the twice-daily arm vs 10% in the once-daily arm)^22^; although the *P* value was not reported, the rate in the group that received twice-daily prophylactic cranial irradiation was obviously higher in terms of numerical value.

The timing of prophylactic cranial irradiation was controversial. Data from Lee et al^23^ showed the trend that the overall incidence of brain metastases was higher in the group that received late prophylactic cranial irradiation (offered irradiation after 5-6 courses of chemotherapy) than in the group that received early irradiation group (offered after 2-3 courses of chemotherapy) (23.6% vs 14.3%; *P* = .08). There was no difference in overall survival between the 2 groups. The pooled analysis conducted by Schild et al^24^ also showed that the timing was not associated with subsequent survival across all patients (HR, 1.00; 95% CI, 0.99-1.01; *P* = .76). According to Aupérin et al,^25^ via classifying the interval between the initiation of induction therapy and prophylactic cranial irradiation into less than 4 months, 4 to 6 months, and longer than 6 months, they identified a trend toward a reduction in the rate of brain metastasis with earlier prophylactic cranial irradiation after the initiation of chemotherapy without an overall survival difference. Sas-Korczyńska et al^26^ found that early prophylactic cranial irradiation (performed during chemoradiotherapy) was more effective compared with irradiation applied after combined therapy, which decreased the rate of brain metastasis from 20% to 7.3% (*P* = .009). However, the prophylactic administration of cranial irradiation concurrent with systemic therapy inevitably increased the risk of neurotoxic effects and hematotoxic effects.^10,27,28^ Some physicians do not like to apply prophylactic cranial irradiation too early because they think that prophylactic cranial irradiation decreases the rate of brain metastasis via eliminating micrometastases that cannot be detected by radiologic methods, rather than preventing metastases.^29^ Undergoing prophylactic cranial irradiation too early attenuates the effects since micrometastases have not developed yet. In our study, early prophylactic cranial irradiation did not decrease the rate of brain metastasis significantly compared with late irradiation, but it significantly shortened overall survival and progression-free survival. In line with the National Comprehensive Cancer Network guideline,^1^ our data support that prophylactic cranial irradiation should be administered after the resolution of the acute toxic effects of initial chemoradiotherapy.

Previous studies have shown that the BED and SER may be associated with overall survival^10,30,31^; we also found that the BED was lower and the SER was shorter in the TDRT group in our study. However, the multivariable analysis that jointly evaluated BED, SER, and TDRT and ODRT showed that BED and SER had no significant association with outcomes, which further supports the hypothesis that the observed difference in the incidences of brain metastases may be attributed to the frequency of radiotherapy.

In our study, the lower incidence of brain metastases in the ODRT group did not translate into improved overall survival in the whole cohort. In fact, such an observation is not rare and has been reported before,^32,33,34^ which could be explained by the potential confounding of effective subsequent therapies with higher treatment-related financial costs.^35^ However, after matching with balanced cases, the longer overall survival in the ODRT group became marginally significant, which further indicates that the effect of ODRT vs TDRT is of clinical significance.

In addition, our data showed that patients receiving ODRT lived longer than patients in previous studies.^36^ The longer overall survival resulted in a higher long-term incidence of brain metastases and might also have contributed to the observation of the significant difference in the incidence of brain metastases in our series. A possible cause for the longer overall survival in our study may be that all our patients were Chinese, whereas less than 1% of patients in the CONVERT trial^36^ were of Asian origin. Faivre-Finn et al^36^ also discussed that their results might not be applicable to other races/ethnicities. It is not unusual that different races/ethnicities may show different responses to the same treatment regimen. A case in point is that tyrosine kinase inhibitors are effective for Chinese patients but are less effective for European or American patients^37,38^ because there are more Chinese patients with an epidermal growth factor receptor mutation. In addition, our patients were younger with fewer smokers, which was in line with official Chinese data.^39,40^ According to Jia et al,^39^ among the enrolled 14 106 male patients, 11 750 (83.3%) were smokers. In another study that included 3320 patients, 2223 (67.0%) were smokers.^40^ Other reasons were briefly discussed in an earlier study.^4^

In summary, TDRT was introduced decades ago to clinical practice based on radiobiological principles.^41^ It is time to review real-world experiences and reconsider its value. Our findings may shed light on the benefit of TDRT with the use of the longest feasible interfraction interval^20^ (eg, 12 hours between fractions in the twice-daily regimen). Benefit may even be seen in stopping the use of TDRT for patients with small cell lung cancer, especially in China or other parts of Asia where twice-daily irradiation is logistically less preferred compared with once-daily irradiation because more and more ODRT schedules have shown noninferior outcomes.^42,43,44^

### Limitations

This study has several limitations. First, owing to the retrospective nature of this study, a fraction of patients with missing data had to be excluded from our analysis, which may have led to bias (such as incorrect estimates of difference between types of radiotherapy), limited the generalizability of our findings, and reduced the power to detect clinically meaningful differences in clinical outcomes. Second, despite the fact that we made significant efforts to minimize the potential selection biases using regression and propensity score matching, the current analysis could still be subject to unobserved confounding. Third, the centers are not balanced even after matching. However, considering that patients from the same institution had the same treatment-related or follow-up policy and that the detection rate of asymptomatic brain metastasis via imaging is similar between the ODRT group and the TDRT group in both the whole cohort and the matched cohort, we do not think centers would affect the results. In fact, our data showed that there was only very limited variability in brain metastasis rates across centers.

## Conclusions

In this study, compared with thoracic ODRT, TDRT appears to be associated with a higher risk of brain metastases after prophylactic cranial irradiation in Chinese patients with small cell lung cancer. These findings may motivate more in vitro and in vivo research to further investigate the underlying mechanisms and may also affect the clinical option of the thoracic radiotherapy schedule, especially in China or other parts of Asia.

## Notes

Supplement. **eTable 1.** Thoracic Radiotherapy Dose Details **eTable 2.** Brain Metastases Rate in ODRT and TDRT Groups (N = 338) **eTable 3.** Survival Analysis With ODRT/TDRT, BED and SER (N = 338) **eTable 4.** Brain Metastases Rate in ODRT and TDRT by Stratum of Stage (N = 338) **eTable 5.** Brain Metastases Rate in ODRT and TDRT by Stratum of Diagnosis Year (N = 338) **eTable 6.** Brain Metastases Rate in Earlier and Recent Years by Stratum of ODRT vs TDRT (N = 338) **eTable 7.** Brain Metastases Rate in ODRT and TDRT by Stratum of Early PCI vs Late PCI (N = 338) **eFigure.** Overall Survival and Progression-Free Survival in ODRT Group and TDRT Group (N = 338) **eAppendix.** Follow-up Strategy
