# Supplementary material for: Association of Twice-Daily Radiotherapy With Subsequent Brain Metastases in Adults With Small Cell Lung Cancer
Source: JAMA Netw Open. 2019 May 17;2(5):e190103. doi: 10.1001/jamanetworkopen.2019.0103 (PMC6537825; doi:10.1001/jamanetworkopen.2019.0103)
Supplement: Supplement. — eTable 1. Thoracic Radiotherapy Dose Details eTable 2. Brain Metastases Rate in ODRT and TDRT Groups (N = 338) eTable 3. Survival Analysis With ODRT/TDRT, BED and SER (N = 338) eTable 4. Brain Metastases Rate in ODRT and TDRT by Stratum of Stage (N = 338) eTable 5. Brain Metastases Rate in ODRT and TDRT by Stratum of Diagnosis Year (N = 338) eTable 6. Brain Metastases Rate in Earlier and Recent Years by Stratum of ODRT vs TDRT (N = 338) eTable 7. Brain Metastases Rate in ODRT and TDRT by Stratum of Early PCI vs Late PCI (N = 338) eFigure. Overall Survival and Progression-Free Survival in ODRT Group and TDRT Group (N = 338) eAppendix. Follow-up Strategy [file jamanetwopen-2-e190103-s001.pdf]

Supplementary Online Content

Zeng H, Li R, Hu C, et al. Association of twice-daily radiotherapy with subsequent brain metastases in adults with small cell lung cancer. *JAMA Netw Open.* 2019;2(5):e190103. doi:10.1001/jamanetworkopen.2019.0103

eTable 1. Thoracic Radiotherapy Dose Details
eTable 2. Brain Metastases Rate in ODRT and TDRT Group (N=338)
eTable 3. Survival Analysis With ODRT/TDRT, BED and SER (N=338)
eTable 4. Brain Metastases Rate in ODRT and TDRT by Stratum of Stage (N=338)
eTable 5. Brain Metastases Rate in ODRT and TDRT by Stratum of Diagnosis Year (N=338)
eTable 6. Brain Metastases Rate in Earlier and Recent Years by Stratum of ODRT vs TDRT (N=338)
eTable 7. Brain Metastases Rate in ODRT and TDRT by Stratum of Early PCI vs Late PCI (N=338)
eFigure. Overall Survival and Progression-Free Survival in ODRT Group and TDRT Group (N=338)
eAppendix. Follow-up Strategy

This supplementary material has been provided by the authors to give readers additional information about their work.

© 2019 Zeng H et al. *JAMA Network Open.*

eTable 1. Thoracic Radiotherapy Dose Details

Groups	Schedules	No.	Constituent ratio (%)
ODRT			
	50-66Gy/25-33F	569	93.4
	>66Gy	6	1.0
	<50Gy	34	5.6
	Total	609	100
TDRT			
	45Gy/30F/bid	145	85.8
	>45Gy*	23	13.6
	<45Gy**	1	0.6
	Total	169	100

Abbreviations: ODRT = once-daily radiotherapy; TDRT = twice-daily radiotherapy.

Note: *Including 9 patients with late course accelerated hyperfractionated radiotherapy; **37.5Gy/25F/bid

© 2019 Zeng H et al. *JAMA Network Open.*

eTable 2. Brain Metastases Rate in ODRT and TDRT Group (N=338)

Events		Cumulative Rate (95%CI) - %					*P* value
		1-yr	2-yr	3-yr	4-yr	5-yr	(Gray's test)
Competing event*							
	ODRT	2.0 (0.5-5.2)	16.5 (10.9-23.2)	25.8 (18.2-34.1)	38.0 (27.5-48.6)	40.7 (29.3-51.8)	0.83
	TDRT	8.3 (4.7-13.4)	20.9 (14.6-28.0)	25.6 (18.2-33.6)	29.3 (20.7-38.3)	29.3 (20.7-38.3)	
Brain metastases							
	ODRT	3.8 (1.6-7.7)	12.2 (7.5-18.1)	14.9 (9.5-21.4)	17.0 (10.6-24.7)	20.5 (11.8-31.0)	0.04
	TDRT	9.6 (5.6-14.8)	21.4 (15.1-28.6)	26.0 (18.6-34.0)	28.1 (19.9-36.9)	28.1 (19.9-36.9)	

Abbreviations: ODRT = once-daily radiotherapy; TDRT = twice-daily radiotherapy

Note: *Competing event includes death without brain metastases.

© 2019 Zeng H et al. *JAMA Network Open.*

eTable 3. Survival Analysis With ODRT/TDRT, BED and SER (N=338)

Endpoints		Univariate				Multivariate		
		P Value	HR	95%CI		*P* Value	HR	95%CI
Brain metastases*								
	ODRT/TDRT	0.04	1.71	1.02-2.88		0.03	1.98	1.09-3.59
	BED	0.44	0.99	0.95-1.02		0.45	1.02	0.97-1.06
	SER	0.95	1.00	1.00-1.01		0.58	1.00	1.00-1.01
Progression-free survival**								
	ODRT/TDRT	0.30	1.16	0.87-1.55		0.31	1.20	0.84-1.72
	BED	0.41	0.99	0.97-1.01		0.86	1.00	0.97-1.03
	SER	0.18	1.00	1.00-1.01		0.11	1.00	1.00-1.01
Overall survival**								
	ODRT/TDRT	0.06	1.41	0.99-2.01		0.03	1.69	1.05-2.71
	BED	0.57	0.99	0.96-1.02		0.37	1.02	0.98-1.06
	SER	0.31	1.00	1.00-1.01		0.14	1.00	1.00-1.01

Abbreviations: ODRT = once-daily radiotherapy; TDRT = twice-daily radiotherapy; BED = biologically effective dose; SER = Start of any therapy to the end of radiotherapy; HR = hazard ratio; CI = confidence interval .

Note: *Fine-Gray model; **Cox regression model

© 2019 Zeng H et al. *JAMA Network Open.*

**eTable 4.** Brain Metastases Rate in ODRT and TDRT by Stratum of Stage (N=338)								
Subgroups			BM rate (%)			Fine and Gray model		
		No. (Ratio)	3-yr	5-yr		*P* Value	HR	95% CI
Limited disease								
	ODRT	143 (49.8)	14.3	20.1		0.08	1.67	0.94 - 2.96
	TDRT	144 (50.2)	24.6	26.7				
	All limited	287 (84.9)*	20.4	NA		0.15*	1.65*	0.84 - 3.23*
Extensive disease								
	ODRT	26 (51.0)	20.3	NA		0.29	1.91	0.58 - 6.32
	TDRT	25 (49.0)	33.5	NA				
	All extensive	51 (15.1)*	26.8	NA				
Adjusting by stage**						0.04	1.71	1.02 - 2.87

Abbreviations: ODRT = once-daily radiotherapy; TDRT = twice-daily radiotherapy; NA = non-applicable.
Note:* Compare between limited and extensive disease; ** Compare between ODRT and TDRT.

© 2019 Zeng H et al. *JAMA Network Open.*

eTable 5. Brain Metastases Rate in ODRT and TDRT by Stratum of Diagnosis Year (N=338)

Subgroups			BM rate (%)			Fine and Gray model		
		No. (Ratio)	3-yr	5-yr		*P* Value	HR	95% CI
Earlier years (2003-2010)								
	ODRT	30 (51.7)	14.2	19.5		0.03	3.05	1.09 - 8.53
	TDRT	28 (48.3)	45.2	45.2				
	All earlier	58 (17.2)*	28.7	31.4		0.12*	0.63*	0.35 - 1.13*
Recent years (2011-2016)								
	ODRT	139(49.6)	15.1	20.6		0.21	1.47	0.80 - 2.68
	TDRT	141(50.4)	21.8	25.0				
	All recent	280 (82.8)*	18.3	22.8				
Adjusting by earlier/recent years**						0.03	1.78	1.05 - 3.00

Abbreviations: ODRT = once-daily radiotherapy; TDRT = twice-daily radiotherapy; NA = non-applicable.
Note:* Compare between earlier and recent years; ** Compare between ODRT and TDRT.

© 2019 Zeng H et al. *JAMA Network Open.*

**eTable 6.** Brain Metastases Rate in Earlier and Recent Years by Stratum of ODRT vs TDRT (N=338)								
Subgroups			BM rate (%)			Fine and Gray model		
		No. (Ratio)	3-yr	5-yr		*P* Value	HR	95% CI
ODRT								
	Earlier years	30 (17.8)	14.2	19.5		0.95	0.97	0.37 - 2.55
	Recent years	139 (82.2)	15.1	20.6				
	All ODRT	169 (50.0)*	14.9	20.5		0.04*	1.71*	1.02 - 2.88*
TDRT								
	Earlier years	28 (16.6)	45.2	45.2		0.04	0.46	0.22 - 0.96
	Recent years	141 (83.4)	21.8	25.0				
	All TDRT	169 (50.0)*	26.0	28.1				
Adjusting by ODRT/ODRT**						0.10	0.61	0.34 - 1.10

Abbreviations: ODRT = once-daily radiotherapy; TDRT = twice-daily radiotherapy; NA = non-applicable.

Note:* Compare between ODRT and TDRT;

** Compare between earlier (2003-2010) and recent years (2011-2016).

© 2019 Zeng H et al. *JAMA Network Open.*

**eTable 7.** Brain Metastases Rate in ODRT and TDRT by Stratum of Early PCI vs Late PCI (N=338)								
Subgroups			BM rate (%)			Fine and Gray model		
		No. (Ratio)	3-yr	5-yr		*P* Value	HR	95% CI
Early PCI								
	ODRT	26 (44.1)	28.2	28.2		0.54	1.39	0.49 - 3.99
	TDRT	33 (55.9)	32.3	32.3				
	All early	59 (17.5)*	30.2	30.2		0.12*	1.58*	0.89 - 2.84*
Late PCI								
	ODRT	143 (51.3)	12.8	19.5		0.05	1.81	0.99 - 3.31
	TDRT	136 (48.7)	24.4	28.0				
	All late	279 (82.5)*	17.9	30.2				
Adjusting by early/late PCI**						0.05	1.71	1.01 - 2.89

Abbreviations: ODRT = once-daily radiotherapy; TDRT = twice-daily radiotherapy; NA = non-applicable.
Note:* Compare between late PCI and early PCI; ** Compare between ODRT and TDRT.

© 2019 Zeng H et al. *JAMA Network Open.*

eFigure. Overall Survival and Progression-Free Survival in ODRT Group and TDRT Group (N=338)

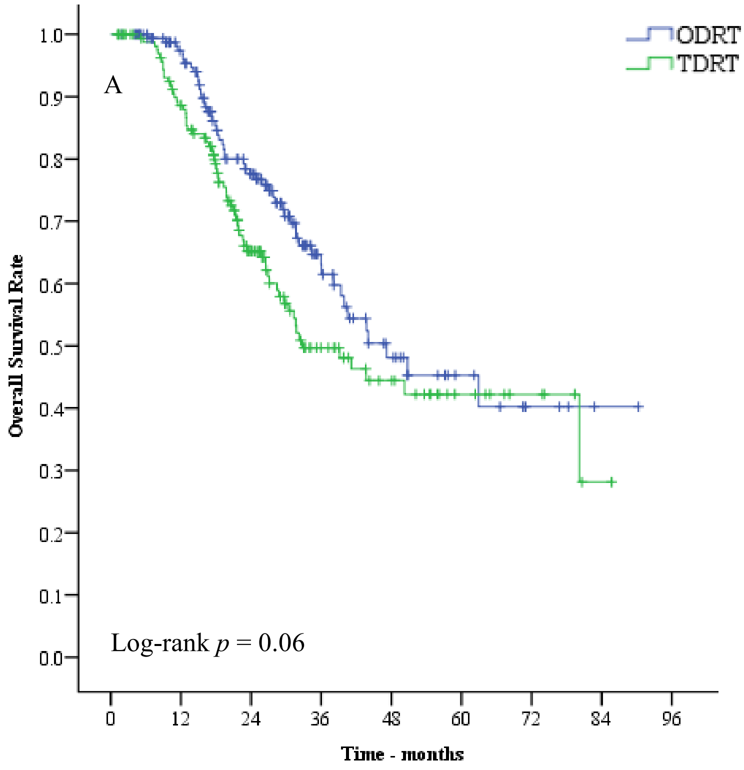

No. at Risk

ODRT	169	144	94	40	21	10	4	1
TDRT	169	137	74	35	22	11	6	1

© 2019 Zeng H et al. *JAMA Network Open.*

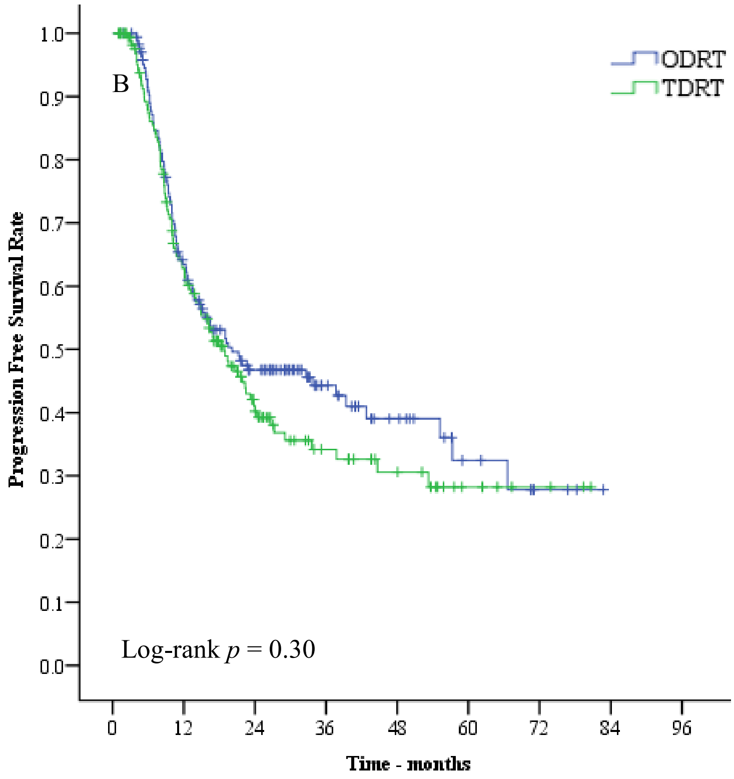

No. at Risk

ODRT	169	100	61	29	17	8	3
TDRT	169	95	43	22	15	6	3

Shown are overall survival rate (Panel A) and progression-free survival rate (Panel B) in two groups. Overall survival tended to be significantly longer in ODRT while progression-free survival was not significantly different between ODRT and TDRT groups.

ODRT, once-daily radiotherapy; TDRT, twice-daily radiotherapy.

© 2019 Zeng H et al. *JAMA Network Open.*

eAppendix. Follow-up Strategy

The follow-up strategy was every month for the first three months, then every three months for the following two years, every six months for the next three years and annually thereafter until death. Investigations included at least taking a medical history and performance status evaluation. Work-up included laboratory test, contrast-enhanced thoracic and abdominal CT scan or more (like PET-CT). Patients with key symptoms of brain metastases such as headache, dizzy, vision damage, nausea, vomit, extremities motionless had to be performed with contrast-enhanced CT or MRI of the head. Otherwise clinician specified whether perform it or not on the basis of patients' willingness. Patients could visit our institutes or the local institutes at their convenience for follow-up. And we interviewed all the patients or their caregivers by telephone at least twice to confirm their healthy status and quality of life (QoL). Patients were treated as lost to follow up if we failed to contact them or their caregivers. The follow-up data were cutoff November, 2017.

Of the 778 eligible patients, 157(19.9%) patients were lost to follow-up (123 [20.2%] from ODRT and 34 [20.1%] from TDRT, p = 0.98). For the whole 778 patients, the median follow-up time was 23.6 months (IQR, 14.2-38.2 months). For the 621 patients who followed up until November 2017, the median time was 26.5 months (IQR, 17.5-41.2 months). For the 157 patients lost to follow up, the median time was 12.0 months (IQR, 5.7-21.3 months). In the matched cohort with 338 patients, 74 (22.5%) lost to follow-up (40 [23.7%] were from

© 2019 Zeng H et al. *JAMA Network Open.*

ODRT and 34 [20.1%] from TDRT, $p = 0.430$). For the whole 338 patients, the median follow-up time was 23.9 months (IQR, 15.5-34.3 months). For the 264 patients who followed up until November 2017, the median follow-up time was 26.4 months (IQR, 17.7-38.1 months). For the 74 patients lost to follow up, the median follow-up time was 13.4 months (IQR, 5.5-24.3 months).

© 2019 Zeng H et al. *JAMA Network Open.*
